# Phytotoxic Effects of (±)-Catechin *In vitro*, in Soil, and in the Field

**DOI:** 10.1371/journal.pone.0002536

**Published:** 2008-07-02

**Authors:** Inderjit, Jarrod L. Pollock, Ragan M. Callaway, William Holben

**Affiliations:** 1 Centre for Environmental Management of Degraded Ecosystems (CEMDE), University of Delhi, Delhi, India; 2 Division of Biological Sciences, The University of Montana, Missoula, Montana, United States of America; University of Pretoria, South Africa

## Abstract

**Background:**

Exploring the residence time of allelochemicals released by plants into different soils, episodic exposure of plants to allelochemicals, and the effects of allelochemicals in the field has the potential to improve our understanding of interactions among plants.

**Methodology/Principal Findings:**

We conducted experiments in India and the USA to understand the dynamics of soil concentrations and phytotoxicity of (±)-catechin, an allelopathic compound exuded from the roots of *Centaurea maculosa*, to other plants *in vitro* and in soil. Experiments with single and pulsed applications into soil were conducted in the field. Experimental application of (±)-catechin to soils always resulted in concentrations that were far lower than the amounts added but within the range of reported natural soil concentrations. Pulses replenished (±)-catechin levels in soils, but consistently at concentrations much lower than were applied, and even pulsed concentrations declined rapidly. Different natural soils varied substantially in the retention of (±)-catechin after application but consistent rapid decreases in concentrations over time suggested that applied experimental concentrations may overestimate concentrations necessary for phytotoxicity by over an order of magnitude. (±)-Catechin was not phytotoxic to *Bambusa arundinacea* in natural Indian soil in a single pulse, but soil concentrations at the time of planting seeds were either undetectable or very low. However, a single dose of (±)-catechin suppressed the growth of bamboo in sand, in soil mixed with organic matter, and *Koeleria macrantha* in soils from Montana and Romania, and in field applications at 40 µg l^−1^. Multiple pulses of (±)-catechin were inhibitory at very low concentrations in Indian soil.

**Conclusions/Significance:**

Our results demonstrate that (±)-catechin is highly dynamic in natural soils, but is phytotoxic well below natural concentrations measured in some soils and applied at low concentrations in the field. However, there is substantial conditionality in the effects of the allelochemical.

## Introduction

Allelopathic effects have been attributed to a number of exotic invasive plants [1] and recent research has also suggested the possibility that some invaders may possess novel chemicals that are more phytotoxic to naïve and non-adapted native plants, soil microbes, or herbivores in the invaded range than adapted species in the invader's native range [2]–[8]. Such biogeographical differences in the effects of phytotoxic, antimicrobial, or defense biochemistry have been proposed as a mechanism for invasion - the “Novel Weapons Hypothesis” [9]–[11]. In this context, the allelopathic effects of the North American invasive weed, *Centaurea maculosa* Lam. [spotted knapweed, recently suggested to be *C. stoebe* L. (USDA, NRCS 2007)], have been studied extensively.

Phytotoxic effects have been reported from *C. maculosa* leaves [12] and roots [13] and potentially biologically-active compounds isolated from species in the *Centaurea* genus include aromatic amines, chromenes, phenols, nonterpenoid lactones, lignins, and triterpenes [14], [15]. Also, phytotoxic effects of (±)-catechin, a phenolic compound exuded from the roots of *C. maculosa* (or the separated forms of (−) or (+) catechin), have been demonstrated *in vitro*, in sand culture, in controlled experiments with field soils, and in the field [6], [16]–[27] (J. Pollock and W. Holben, *unpublished results*). (±)-Catechin has also been implicated in the Novel Weapons Hypothesis [18] (W. He & R.M. Callaway, unpublished data).

However, seemingly similar experiments have not always shown (±)-catechin to have inhibitory effects for all species [22], [28]. Also, different soil types have been reported to eliminate phytotoxicity of (+)-catechin (one isomer of the (±) combination [25]), and inhibitory effects vary substantially among target plant species [6], [22], [23]. Furthermore, field application of (±)-catechin to soils at the same site and to the same plant species show substantial variation between years (6,22). Even within the same growing season, application of (±)-catechin dramatically reduced the growth of *Geum triflorum* Pursh in open grassland [6], but the same concentration had no detectable effect on *G. triflorum* in soils under *Pseudotsuga menzeisii* tree canopies several meters away (G.C. Thelen & R.M. Callaway, *unpublished results*).

Most importantly, whether or not natural soil concentrations of (±)-catechin reach phytotoxic concentrations is questionable. Recent measurements show that concentrations are usually far lower than initially reported, and vary much more spatially and temporally in *C. maculosa* rhizospheres than earlier reported [28]–[30]. Recent extensive sampling of soil catechin concentrations recorded a mean of 650±450 µg g^−1^ (1 SD), with 20 out of 20 rhizospheres containing catechin) at one site and at one time in the growing season, but at the same site over six other sampling periods no (±)-catechin was detected, but using an approach with a detection limit of 25 µg g^−1^ [30] This raises the possibility that (±)-catechin may be released in pulses. At 10 other sites that were sampled only once, but at other times, no (±)-catechin was detected in *C. maculosa* rhizospheres [30]. Other sampling efforts have detected (±)-catechin in soils more frequently in *C. maculosa* rhizospheres, but at far lower levels, ranging from 0–1 µg g^−1^ [29]. These results also suggest that (±)-catechin may be more abundant at some times during the growing season than others. It is important to note that bulk soil concentrations such as these suggest target concentrations for soil experiments, but are not relevant for estimating the phytotoxicity of experimental solutions. This is in part because most soil sampling and analytical techniques result in an “averaging” of the measured concentration of the chemical in bulk soil, not in the soil solution, and the actual spatial distribution of the chemical is likely to be concentrated at the rhizoplane of the exuding roots. Experimental concentrations for (±)-catechin solutions may also be estimated by the concentration of (±)-catechin achieved by root exudation from *C. maculosa* into solution. For seedlings this has been reported at 0–2.4 µg ml^−1^ [28], 5–35 µg ml^−1^ [19]–[20], 0–113 µg ml^−1^ [31] and 83–185 µg ml^−1^ [17]. These concentrations should be considered rough estimates as in some of these cases the solution was designed to stabilize this highly dynamic chemical, seedlings were not exposed to natural light or natural soils, and seedling exudation may not be comparable to adult exudation.

Variation in the environmental concentrations of a putative allelopathic chemical, and in the results of experimental tests for its toxicity, could be due to many other reasons: use of different experimental chemical concentrations, subtle differences in the age or nature of the chemical applied, natural instability of the chemical *in vitro* or *in situ*, the age, size, or health of the target plants, seasonal timing of experimental application or soil collection, soil temperature or moisture, or different analytical or methodological techniques. Furthermore, most plants do not continuously biosynthesize secondary metabolites throughout their life cycle and allelopathic compounds have been shown to be dynamic in soils [32], [33] and in their production [34]. The extreme variation reported for soil (±)-catechin concentrations is probably also affected by oxidation of the molecule, which results in changes in its absorption spectrum [25] and the exudate's chelating effects on metals to potentially release insoluable phosphates [35]. These factors almost certainly cause experimental concentrations in solutions and substrates to be far lower than those initially applied [36]–[38], resulting in substantial mismatches between applied experimental and *in situ* concentrations. Furthermore, although single dose experiments are a crucial step towards understanding the effects of putative allelochemicals in natural settings and may mimic pulsed releases, applying single doses of a biochemical to soil is likely to underestimate the concentration of the chemicals released as exudates over long periods of time.

The possibility of pulsed deliveries of root exudates [30] raises other questions about experimental protocol. The concentrations of biologically active chemicals that can be detected and measured in soil may vary periodically as plants sporadically vary in the exudation of chemicals (32]. Temporal fluxes in the concentration of allelopathic chemicals have been measured in soils [33], temporal dynamics have been measured in production [34], and temporal effects have been modeled [39], but to our knowledge no studies have attempted to mimic such dynamics in experimental applications.

We focus on three aspects of the effects of (±)-catechin that require better resolution: 1) matching exuded concentrations and measured concentrations in soils to phytotoxicity, 2) determining the potential for low concentrations of (±)-catechin in soil to be phytotoxic, and 3) measuring the fate and effect of pulsed, or dynamic, experimental deliveries. We tackled these issues by conducting Petri dish assays using lower concentrations than tested in the past, making repeated measurements of (±)-catechin in soil after experimental applications, and testing the phytotoxicity of (±)-catechin in different soils, different application concentrations, in pulsed applications, and in the field.

## Materials and Methods

### In vitro phytotoxicity tests

We first tested the potential of low *in vitro* concentrations of (±)-catechin to be phytotoxic to our two focal test species, *Bambusa arundinacea* (bamboo), which is native to India and *Koeleria macrantha* (Ledeb.) J.A. Schultes, a species native to North America. We chose *Bambusa* because it is highly abundant in subtropical India and appears to be a dominant competitor in its native range. We chose *Koeleria* through a random draw from the names of 6 dominant native Montana grass species placed in a hat. *Bambusa* is from a region in which *C. maculosa* does not occur. *Koeleria* occurs in invaded grasslands and we used field collected seeds from areas with low densities of *C. maculosa*. For each species, 10 seeds were placed on Whatman # 1 filter paper in each of 15 9-cm diameter Petri dishes. For each species, in 5 of these Petri dishes the filter papers were initially wetted with 10 ml of a catechin solution (50 mg of (±)-catechin dissolved in 10 ml of 100% methanol and then diluted in 990 ml HPLC-pure water for a final 50 µg ml^−1^). In 5 other Petri dishes for each species 10 ml of a 25 µg ml^−1^ solution was applied. In another 5 Petri dishes for each species only the water/methanol (99∶1 v/v) solution was applied. Several similar studies have demonstrated (±)-catechin phytotoxicity for many different species at higher concentrations than we used [21], [40], but we chose these lower concentrations because they were similar to that reported in a recent seedling exudation experiment (Ridenour *et al.*, *in press* [31]) and between the concentrations reported by Bais *et al.* [16], Weir *et al.* [19]–[20] and Blair *et al.* [28]. Petri dishes were sealed with Parafilm, kept at 21–23°C and exposed to a 12∶12 hour day∶night schedule. Root length was measured 12 days after the application of the treatments to the seeds, and treatments were compared for each species using ANOVA and post ANOVA Tukey tests (n = 5).

### Soil concentrations

The potential for (±)-catechin to be phytotoxic at natural soil conditions has been the most controversial aspect of *C. maculosa* allelopathy, and as discussed above the most difficult to test. Recent extensive measurements show clearly that earlier studies dramatically overestimated natural soil concentrations [29], [30], pure (±)-catechin diminishes very rapidly in soils immediately after application [28], and (±)-catechin appears to occur in *C. maculosa* rhizospheres in the field in episodic pulses [30]. This creates experimental problems. Applied amounts dramatically overestimate the longer term resident concentrations in soils that target plants experience, but applying the concentrations necessary to achieve low soil concentrations during the course of a multi-day experiment requires exposing target plants to pulses of very high concentrations at the time of application. Such pulses may be experienced in nature, but it is difficult to estimate how often they occur, or the concentrations experienced by the root surfaces of neighboring plants. We approached this problem by combining different experimental approaches including planting seeds well after (±)-catechin application, measuring bulk concentrations in our experimental soils, and conducting experiments in which seeds and seedlings either experienced pulses or did not.

The first two experiments (single pulse and multiple pulses, Indian soil) were conducted in parallel at the University of Delhi and The University of Montana with Indian soil and *Bambusa arundinacea* (bamboo). India does not have a history of *C. maculosa* invasion. We therefore assume that soil communities in Indian soil and native Indian plants (e.g. *Bambusa*) have not experienced (±)-catechin exuded from *C. maculosa* roots. The effects of (±)-catechin on *Bambusa* were measured at the University of Delhi. Because the equipment to measure (±)-catechin does not exist at the University of Delhi, soil collected at the same place and time was sent to The University of Montana to explore the relationship between applied (±)-catechin concentrations and extant concentrations in the soil using the same protocol, replication, pots, and experimental conditions in which phytotoxicity was tested. We also explored (±)-catechin dynamics and phytotoxicity in soils from Montana and Romania, and our general intent was simply to ascertain generality, or conditionality, using many different soils.

Blair *et al.* [29] used an approach to measure (±)-catechin in soil that was sensitive to low concentrations and often found (±)-catechin in *C. maculosa* rhizospheres, but never more than 1 µg g^−1^ soil. Perry *et al.* [30], used an approach with a higher detection limit of 25 µg g^−1^ soil and found detectable levels of (±)-catechin far less frequently, but reported a pulse at a repeatedly measured site averaging 650±450 µg g^−1^ (1 SD). Previous studies reported higher and more consistent soil concentrations of (±)-catechin, but as discussed in [30], we do not consider those measurements to be accurate. In experiments we targeted resident soil concentrations at 0–50 µg g^−1^.

To measure (±)-catechin, 1 g soil was collected in sterile Eppendorf tubes, amended with 1 ml of 100% methanol, briefly mixed by vigorous vortexing, pelleted by centrifugation for 10 min at 13000 rpm, and the supernatant was placed into vials for HPLC analysis [41]. In brief, catechin concentrations were measured by high-pressure liquid chromatography (HPLC) using 15 µL injection volumes with UV detection at 280 nm on a HP series 1100 with a HP ODS Hypersil C18 column (5 µm, 125×4 mm) using a methanol (25% v/v) -phosphoric acid (15 mM) mobile phase at a flow rate of 1 ml/min. This method employs an isocratic elution, which potentially eliminates variability among peak areas that occurs when employing an increasing gradient elution as in other methods [29], [42]. Also, this technique's limit of detection ranged from 5 µg ml^−1^ to 3000 µg ml^−1^ of (±)-catechin for standard solutions dissolved in 100% methanol immediately after preparation, and is therefore an effective methodological approach. Thus, differences measured in applied concentrations of (±)-catechin and concentrations of (±)-catechin in soils are most likely due to transformation of the pure form through chelation, sorption, oxidation, microbial processes, or other unknown soil effects.

### Single pulse, Indian soil

In India we collected soil from under native vegetation (sandy loam; pH, 7.7; organic matter, 0.95%) of Delhi, India (Lat., 28.38 N; Long., 77.12 E). This soil has no history of exposure to any *Centaurea* species. For each of 6 replicates per treatment, 150 g soil was added to a 190 cm^3^ pot (n = 6 for each treatment) which then was treated with 40 ml of 0 µg, 500 µg, 1000 µg or 1500 µg ml^−1^ (±)-catechin dissolved in 100% methanol, then diluted to 5% methanol in HPLC-grade water (v/v) designed to apply concentrations of 0 µg, 133 µg, 266 µg and 400 µg (±)-catechin g^−1^ soil.

### Multiple pulses, Indian soil

We dissolved (±)-catechin in 100% methanol and then further diluted it into HPLC-pure water (5∶95 v/v) to obtain final concentrations of 0, 500, 1000 and 1500 µg ml^−1^. Ten replicates of 50 g soil (the same as in the first experiment) in 50 ml vials were initially treated with 15 ml (day 1) of (±)-catechin (at the 0, 500, 1000 and 1500 µg ml^−1^ concentrations), and then soils were irrigated three times (days 3, 7, 10) with 5 ml of the appropriate concentration, and one time with 4 ml (day 14) of the solutions. This established treatments in which all replicated vials received 34 ml of solution, and in which a total of 0, 340, 680, or 1020 µg g^−1^ of (±)-catechin was added to the soil. This application rate saturated the soils with the solutions, but did not leave solution standing on top of the soils. The tubes were incubated under alternating 12-hr light and 12-hr dark period at 22–24°C for 17 days. To determine the stability and maximum accumulated *in situ* soil concentrations achieved by these applications, we sampled immediately after application, on days 3 and 7, both before and after the day 10 and day 14 applications, and again on day 17. To measure (±)-catechin, a 1 g soil sample was collected from each tube for each measurement and processed for HPLC analysis as outlined above.

### Single pulses, North American and European soils

To establish soil concentrations for other experiments we also tested the stability of (±)-catechin in soils where *C. maculosa* currently occurs, and measured variation in the retention of a single experimentally applied pulse of the compound among soils from different sites. We collected soils from grasslands in which *C. maculosa* was present, but from the rhizospheres of the most abundant native grasses. Soil samples were collected from 5 sites in Romania, where *C. maculosa* is native, and 5 sites in Montana where *C. maculosa* is an aggressive exotic invader. The locations of the Romanian sites were at 47.13 N/26.29 E, 46.5 W, 26.56 E, 47.09 N/27.35 E, 47.10 N/22.52 W, 45.51 N/27.26 E and the locations of the Montana sites were at 46.35 N/112.04 W, 46.51 N/113.59 W, 46.60 N/113.57 W, 46.10 N/113.46 W and 48.52 N/115.03 W.

For each of these 10 sites, we placed 50 g soil into five 50 ml vials and treated them with a single dose of (±)-catechin added in 10 ml of HPLC-grade water. We dissolved (±)-catechin into water by gradually warming the solution to ≈80°C and gently stirring. This new approach is promising as methanol-water solutions do not keep (±)-catechin in solution as long as warmed water (J. Pollock, *personal observation*) and methanol may have unknown effects on plants. This solution was concentrated at 2,500 µg ml^−1^ and achieved an initial calculated soil concentration of 500 µg g^−1^. We began with this high concentration because it is close to the mean concentration of the pulse measured by Perry *et al.* [30] and because preliminary experiments indicated that a pulse of this magnitude would be necessary to achieve even trace amounts after several days. Tubes in this experiment were incubated in the dark because sunlight appears to increase the oxidation rate of (±)-catechin (J. Pollock, *personal observation*). Soil concentrations of (±)-catechin were measured immediately after application and after 1, 3 and 10 days of incubation with the methodology described above. We tested the effects of region (fixed), site (random and nested within region) using a separate ANOVA for each time (fixed) on catechin concentration. We performed these tests using the PROC GLM module within SAS using Type III sum of squares (version 9.1).

Using a subset of these soils (two sites) we also extracted (±)-catechin from the same soils using the methanol solution used by Perry *et al.* [22] and the phosphoric acid (0.1% final concentration) solution used by Blair *et al.* [28] to test for the possibility that the methanol extraction methodology was failing to extract large portions of (±)-catechin from the soils. Standards of (±)-catechin were also made following Blair *et al.* [28] for this test.

### Phytotoxicity experiments in soils

#### Single pulses, Indian soil

The first experiment conducted with Indian soil and *Bambusa* was designed to test the effect of single pulses of (±)-catechin, applied at relatively high doses. For each of 6 replicates per treatment, 150 g soil was added to a 190 cm^3^ pot, and different treatments received 40 ml of 0 µg, 500 µg, 1000 µg or 1500 µg ml^−1^ (±)-catechin dissolved in 100% methanol, then diluted to 5% methanol in HPLC-grade water (v/v), which was designed to establish treatment concentrations of 0 µg, 133 µg, 266 µg and 400 µg catechin g^−1^ soil. However, as described in the results these one-time applications resulted in relevant soil treatments for this experiment of undetectable soil concentrations in the control, the 133 µg, and the 266 µg treatments and 60±20 µg g^−1^ in the 400 µg treatment within an hour after addition. Two days later, equivalent to prior to planting *Bambusa* seeds, the 60 µg g^−1^ concentration decreased to a mean concentration of 4.3±2.4 µg g^−1^ (also see the multiple pulse experiment below). Over the course of the experiment 25 ml of HPLC-grade water was added to each pot to maintain soil moisture. In each of these pots 6 *Bambusa* seeds were planted and seedling mass was measured 14 days later. Untransformed seedling mass was tested with a single ANOVA using “treatment” and “pot” as fixed factors and to differentiate among specific treatments a post-ANOVA Tukey test was conducted (significance limit at P<0.01; SPSS 15.0 [43].

When these experiments had ended, the soil treated with (±)-catechin (0, 133, 266 or 400 µg g^−1^ soil) was analyzed for pH, electrical conductivity (EC), organic carbon (OC), exchangeable phosphate-P, total organic nitrogen (N) and total phenolics after air-drying for 24 hours. Five g of soil was soaked with 25 ml water followed by filtration. One aliquot of the soil filtrate was used to measure pH and EC using a pH and conductivity meter (Metrex, 231-R), while a second part of the filtrate was used to determine total phenolics using Folin and Ciocalteu's phenol reagent [44]. (±)-Catechin is a flavonoid and likely to undergo rapid microbial degradation to yield phenolic acids [45]. Thus, the levels of total phenolics in (±)-catechin-treated soil allowed us to examine whether or not direct degradation products of (±)-catechin, other than oxidized or chelated forms, increase with increasing treatment levels. Soil organic carbon was determined using a chromate titration method [46] (Piper 1966). To determine phosphate-P, 5 g soil was soaked with 2.5% acetic acid, shaken for 30 min followed by filtration. Exchangeable phosphate-P was determined using the molybdenum blue method (Allen 1989). To determine total organic N, 1 g soil was digested using the Kjeldahl method, and N concentration was determined using indophenol method [47]. All analyses were done using six replicates of soil.

#### Multiple pulses, Indian soil

A second experiment was conducted with Indian soil and *Bambusa*, designed to test the concentrations achieved by pulsing deliveries and adding larger total quantities of (±)-catechin into the soil over time. In this case, we added 50 g soil to each of twenty-four 85 ml vials and replicates of 6 were repeatedly treated with catechin (as described above) at either 500, 1000 or 1500 µg ml^−1^. An initial application of 15 ml of (±)-catechin at these concentrations was added and then 6 *Bambusa* seeds were sown 2–5 mm below the surface of the treated soil. Plants were then irrigated 3 more times with 5 ml and one time with 4 ml of each concentration of (±)-catechin over a 14 day period, after which shoot height and mass were measured. Soil treated with a total of 34 ml of 0 µg, 500 µg, 1000 µg or 1500 µg ml^−1^ (±)-catechin in solution was equivalent to adding 0 µg (control), 340 µg, 680 µg and 1020 µg catechin g^−1^ soil, but, as described below the measured mean soil concentrations for the course of the experiment achieved were 0, 1.4±1.4 µg (1SE), 14.5±5.8 µg, and 36.1±10.2 µg g^−1^. (±)-Catechin was not applied directly in contact with the seedlings but to the soil surrounding them, but it is important to note that in order to achieve low soil concentrations, target seedlings were briefly exposed to high concentrations of (±)-catechin during the last three applications. As a control, 6 pots received methanol and distilled water instead of the (±)-catechin solution. Untransformed seedling mass was tested with a single ANOVA using “treatment” and “pot” as fixed factors and to differentiate among specific treatments a post-ANOVA Tukey test was conducted (significance limit at P<0.01; SPSS 15.0 [42]).

#### Single pulses, other Indian soils

Based on prior reports [45] we reasoned that soil organic matter and other factors could affect the phytotoxicity of (±)-catechin. For this reason, and simply to test the effects of the root exudate in a variety of conditions, a third set of phytotoxicity experiments was conducted to test the effects of low concentrations of (±)-catechin in other soil types. We experimented with the natural sandy loam soil collected in India but enriched with organic material, and natural river sand with virtually no organic matter. In the first experiment, compost (native tree litter was composted for 1 year) was added to the original Indian soil to obtain an organic matter content of 1.5% and the mixture was treated with 0, 133, 266 or 400 µg (±)-catechin g^−1^ soil. (±)-Catechin solutions were prepared as described above. In another treatment, 50 g of river sand was amended with the same doses of (±)-catechin. Controls were watered with the methanol-distilled water solution. For controls and treatments, seeds were planted two days after treatments were applied to the soils. For each treatment 6 pots were each planted with 6 bamboo seeds. Growth conditions for these treatments were well lit and had an average day/night temperature of 35/25°C, respectively. After 14 days, shoot height and shoot dry weight were measured. We did not measure (±)-catechin concentrations for this experiment, but all applied concentrations were well below the pulse measured by Perry et al. [29] and measurements reported here for 11 different soil collections indicate that at the time of planting seeds soil concentrations of the compound were far lower than that applied. Statistics were conducted as described for the first two phytotoxicity experiments.

#### Single pulses, North American and European soils

We conducted a fourth phytotoxicity experiment by planting *Koeleria macrantha* seeds into 9 of the 10 soils from Montana and Romania (insufficient soil remained from one of the Romanian sites). The purpose of this experiment was to test for the possibility, suggested in the experiment with the Indian soil, that very low concentrations of (±)-catechin, or (±)-catechin derivatives not evident in measurements of pure (±)-catechin, might also be phytotoxic. This experiment also avoided the problem inherent to the multiple pulse experiment of brief exposure to high concentrations of (±)-catechin at the time of addition. We planted *Koeleria* in the remaining 42 g (8 of the 50 g had been analyzed for (±)-catechin) of soil at the end of the single pulse, North American and European soils, experiment described above. This exposed seeds and germinating seedlings to concentrations below the detectable limit of 5 µg g^−1^ for soil from 7 sites, 10±5 µg g^−1^ for one site and 41±33 µg g^−1^ for one site. In each of the 5 replicate pots per site we planted 10 seeds of *Koeleria* (total treatment n = 45 pots, each with 10 seeds). For each of the 9 sites, we also put 42 g of soil that had not been exposed to (±)-catechin into pots (control n = 3 per site; total n = 27 pots) which were also planted with *Koeleria*. All pots were watered by misting twice per day with tap water for 10 days, after which no new seed emergence was observed. During this period survival of seedlings was recorded and after 10 days pots were no longer watered. Seedling survival was measured for another 11 days after watering ceased. We subjected the seedlings to drought because most *in vitro* experiments for most plant species have shown that the strongest effect of (±)-catechin is on root elongation; not shoot elongation [21]. We hypothesized that inhibition of root growth might expose seedlings to greater suppression by drought, a factor not yet tested in the context of (±)-catechin phytotoxicity. Survival was compared among treatments using the Kaplan-Meier test followed by log rank (Mantel-Cox), Breslow, and Tarone-Ware comparisons (SPSS 15.0, 2006). We also measured the maximum height attained by seedlings in these treatments and tested the effects of region (fixed), site (random and nested within region) in separate ANOVAs using the PROC GLM module within SAS using Type III sum of squares (version 9.1).

#### Single pulse, in situ soils

We conducted a fifth experiment in which we applied (±)-catechin to seedlings in the field at a concentration roughly similar to what accumulates in experimental solutions containing *C. maculosa* seedlings (see above). We created a solution of 40 µg (±)-catechin ml^−1^ water by dissolving it in warm water as described above. Three ml of this solution was injected into the rhizospheres of 15 small *Koeleria macrantha* plants at each of 7 sites in western Montana (46°51′37.22″ N, 113°58′41.24″ W; 46°50′59.75″ N, 113°58′51.82″ W; 46°52′03.83″ N, 113°58′16.45″ W; 46°53′14.42″ N, 113°59′04.80″ W; 46°29′35.83″ N, 114°05′25.25″ W; 46°53′39.74″ N, 113°56′02.84″ W; 46°56′10.91″ N, 113°57′42.20″ W). The 3 ml solution wetted ≈5 g of soil resulting in an estimated initial bulk concentration of ≈24 µg g^−1^. As shown in the results this likely decreased rapidly. Fifteen other plants at each site were treated with 3 ml of water and used as controls. These solutions were injected into soils using a micropipette on 7–8 April 2007, and at this time the number of leaves was counted. On 28–29 April 2007 the number of leaves was counted again, and the change in leaf number was recorded. Change in leaf number was analyzed with ANOVA with treatment as a fixed factor and site as a random factor ((SPSS 15.0, 2006).

## Results

### In vitro phytotoxicity tests

**Figure 1:**
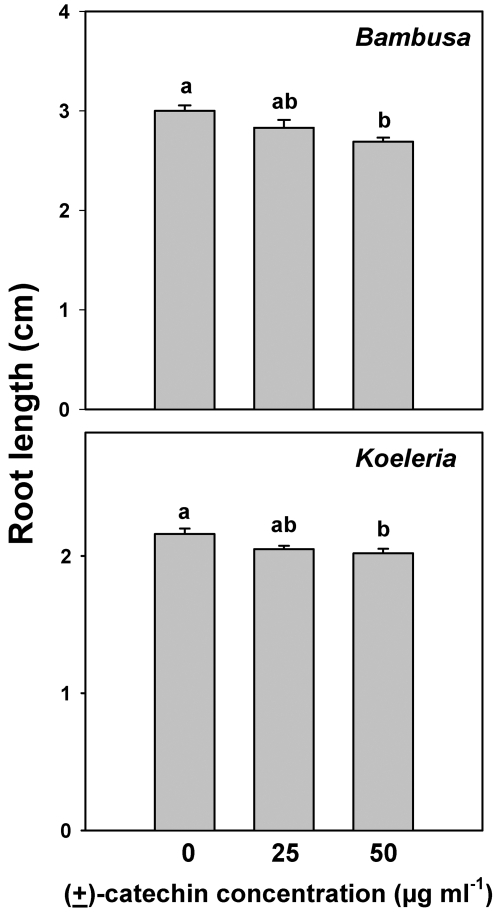
Root length of *Bambusa arundinacea* and *Koeleria macrantha* seedlings exposed to different concentrations of (±)-catechin in Petri dish experiments. Bars indicate 1 SE and shared letters indicate no significant difference among means within a growth measurement as determined by one-way ANOVA and post ANOVA Tukey tests; P<0.05.

(±)-Catechin significantly inhibited the root growth of *Bambusa* and *Koeleria* seedlings at 50 µg ml^−1^ but not at 25 µg ml^−1^ (Fig. 1; ANOVA for *Bambusa*, F_treatment_ = 6.55; df = 2,15; P = 0.012. ANOVA for *Koeleria*, F_treatment_ = 4.97; df = 2,15; P = 0.027). We noted that the (±)-catechin solutions in the Petri dishes appeared to be oxidized (having a red-rust or brown color), indicating that the seedlings in this experiment may have been exposed to concentrations of non-oxidized (±)-catechin that were lower, during most of the duration of the experiment, than the solutions prepared and applied at the onset of the experiment.

### Soil concentrations

#### Single pulses, Indian soil

In the first experiment, single pulse applications of 0 (control) µg, 133 µg, 266 µg and 400 µg g^−1^ of (±)-catechin resulted in soil levels that were not detectable one hour later in the control or the two lowest application rates, and 60±20 (1SE) µg g^−1^ in the highest application rate. Two days later the latter had declined to a mean concentration of 4.3±2.4 µg g^−1^.

#### Multiple pulses, Indian soil

**Figure 2:**
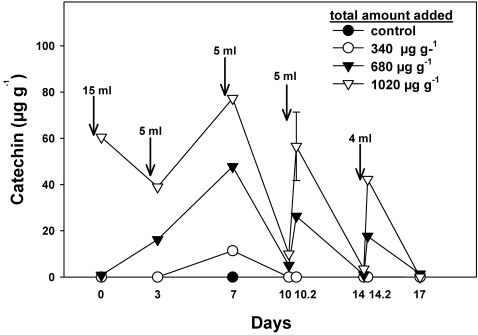
Measured concentrations of (±)-catechin in soil from India, derived from the application of pulsed deliveries of different concentrations shown in the legend. Values on x-axis denote the days on which sampling was conducted. In detail, “10” and “14” denote sampling prior to the application of (±)-catechin pulses on those days, and “10.2” and “14.2” denote sampling two hours after application. The total (±)-catechin delivered to these soils over all pulses was 0, 340, 680, or 1020 µg g^−1^. The single error bar shown indicates the largest 1 SE.

The detected concentrations of (±)-catechin added in repeated pulses to the sandy loam soils from India were also far lower than the applied amounts of (±)-catechin added to the soil (Fig. 2). No (±)-catechin was observed in control soils, and the treatment calculated to add a total of 340 µg (±)-catechin g^−1^ of soil also resulted in zero (±)-catechin detected at all times but one, in which we detected 11.4±4.5 µg g^−1^ immediately after application at day 7. The 680 µg g^−1^ soil (±)-catechin application resulted in a mean of 14.5±5.8 and a maximum of 47.8±20.2 µg g^−1^ immediately after application on day 7. The 1020 µg (±)-catechin application g^−1^ of soil produced a maximum of 77.3±30.9 µg g^−1^ in the soil immediately after application on day 7 and an average across all measurements of 36.1±10.2 µg g^−1^ (±)-catechin in the soil. High soil (±)-catechin concentrations were always associated with measurements taken as soon as possible after application; whereas when measured 3–4 days after application of (±)-catechin, the highest concentration detected was 10 µg g^−1^. Most other measurements at these times were zero. In general, low readings of (±)-catechin corresponded to the development of red-brown coloration of the soil, suggesting that at least a component of the (±)-catechin loss was due to oxidation.

#### Single pulses, North American and European soils

**Figure 3:**
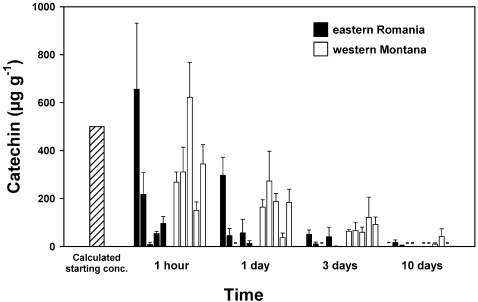
Measured soil concentrations of (±)-catechin applied at 500 µg g^−1^ to soils from western Montana (white bars) and eastern Romania (black bars) and measured over time after application. Error bars show 1 SE. ANOVA statistics are presented in the results.

Soil concentrations measured immediately after application of (±)-catechin in the 5 soils from Montana and the 5 soils from Romania were highly variable, ranging from almost zero for one Montana soil to mean concentrations that were ≈200 µg g^−1^ higher than the calculated application rate of 500 µg g^−1^ (Fig. 3). After 1 day, the mean (±)-catechin concentration for all 10 soils was 126±34 µg g^−1^ soil and the concentration decreased to a range of 0–41±33 µg g^−1^ among the 10 soils 10 days after application. In an ANOVA, the effect of continent (fixed) was not significant (F = 2.298; df = 1,8; P = 0.204); the effect of site (nested, random) was not significant (F = 1.224; df = 8,4; P = 0.424), but the effect of time (fixed) after application was highly significant ((F = 10.535; df = 4,160); P<0.001). The high concentrations early in the time series indicate that our extraction protocol was effective for these soils, and the high degree of variation within sites, and the very high initial spike in one soil, was likely due to the variation in clay and silt fractions in soil and uneven dispersion of the applied (±)-catechin or differences in soil chemistry. The phosphoric acid extraction protocol used by Blair *et al.* [28], with which they reported the highest recovery of experimentally applied (±)-catechin, coupled with our HPLC detection protocol, extracted even less (±)-catechin in the two soils for which we compared the methods. Also, standards made using methods described by Blair *et al.* [28] also demonstrated a decrease in sensitivity for (±)-catechin (data not shown). For soils from Breazu, Romania, the methanol extraction recovered 17.0±10.0 (1 SE) µg g^−1^ 10 days after application, whereas the phosphoric acid and methanol extraction recovered 4.3±2.9 µg g^−1^. For soils from Nelson Gulch, Montana 7.4±7.2 µg g^−1^ (±)-catechin was recovered using the methanol extraction but no (±)-catechin was recovered using the phosphoric acid and methanol extraction.

### Phytotoxicity experiments in soils

#### Single pulses, Indian soil

In the first experiment, in which application rates of 0 (control), 133, 266 and 400 µg g^−1^ of (±)-catechin could not be detected in the control and two lowest application rates, and was measured at 4.3±2.4 µg g^−1^ at the time seeds were planted, we saw no effect of (±)-catechin on the shoot mass of *Bambusa* at any application rate (data not shown).

#### Multiple pulses, Indian soil

**Figure 4:**
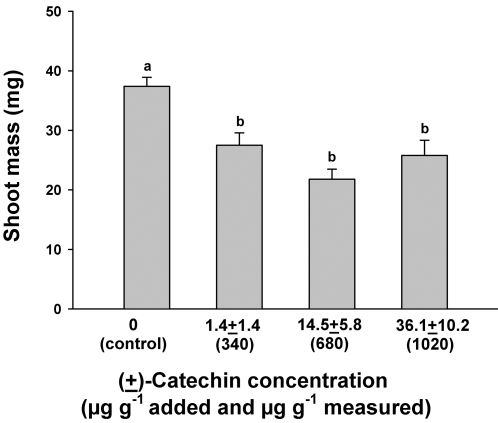
Shoot mass of *Bambusa arundinacea* seedlings exposed to different soil concentrations of (±)-catechin applied in multiple pulses. The x axis shows total measured concentrations of (±)-catechin below the bars and the applied concentrations in parentheses. Bars show 1 SE and shared letters indicate no significant difference among means as determined by ANOVA with treatment and pot as fixed variables and post-ANOVA Tukey tests; P<0.05.

In the second experiment, designed to test the effect of pulsing larger quantities of (±)-catechin into the soil, applications of total amounts of 0 (control), 340, 680 and 1020 µg g^−1^ of (±)-catechin achieved mean measured concentrations over the course of the experiment of 1.4±1.4 (1SE), 14.5±5.8, and 36.1±10.2 µg g^−1^, respectively, in soils (Fig. 3). However, even at these very low soil concentrations, significant phytotoxicity was observed for shoot mass of *Bambusa* at 1.4±1.4, 14.5±5.8, and 36.1±10.2 µg g^−1^ µg g^−1^ (Fig. 4). It is important to note; however, that seeds and seedlings were briefly exposed to higher concentrations of (±)-catechin each time a pulse was added.

The pH of soil treated with 133 (7.99±0.10), 266 (7.65±0.01) or 400 (7.93±0.08) µg catechin g^−1^ soil was significantly higher when compared to untreated (7.23±0.03) soil. The applied concentrations of catechin, however, did not influence conductivity, organic carbon, available phosphorus, or total organic N (data not shown). Even though the detected concentrations of (±)-catechin were far lower than the applied amounts, a significant increase in total phenolics was observed when soil was treated with repeated pulses of (±)-catechin. However, total phenolic concentrations were much lower than would be expected from the total added amounts of (±)-catechin. In control soils total phenolics equaled 3.6±0.3 µg g^−1^; when 133 µg g^−1^ of (±)-catechin was added to the soil 16.2±0.6 µg g^−1^ total phenolics were recovered; when 266 µg g^−1^ of (±)-catechin was added to the soil 23.4±0.4 µg g^−1^ total phenolics were recovered; and when 400 µg g^−1^ of (±)-catechin was added to the soil 36.1±0.5 µg g^−1^ of total phenolics were recovered.

#### Single pulses, other Indian soils

**Figure 5:**
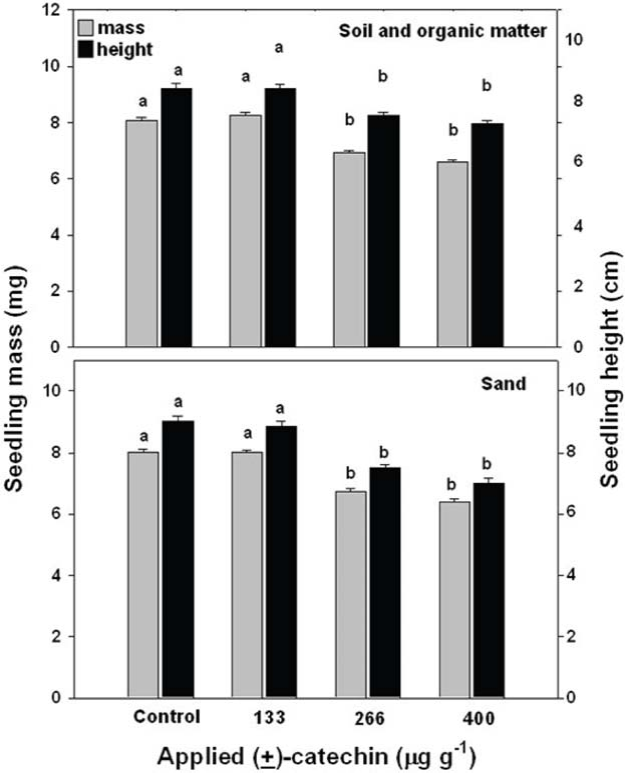
Seedling mass (grey) and height (black) of *Bambusa arundinacea* seedlings planted two days after different concentrations of (±)-catechin were applied to either Indian soil amended with organic matter or natural river sand in a single pulse. Bars show 1 SE and shared letters indicate no significant difference among means as determined by ANOVA with treatment and pot as fixed variables and post-ANOVA Tukey tests; P<0.05.

In the third experiment, in which (±)-catechin was applied to sand or to the Indian soil enriched with organic matter at 0, 133, 266 or 400 µg g^−1^, and seeds were planted two days later, we observed inhibition of shoot mass of *Bambusa* in both substrates at applied rates of 266 µg g^−1^ and higher (Fig. 5). We did not measure (±)-catechin concentrations in the sand or soil plus organic matter treatments but all applied concentrations were lower than the *in situ* soil pulse measured by Perry et al. [30] and results from all other substrates tested here suggest that the amounts of (±)-catechin in these soils at the time of planting was much lower than the applied dose.

#### Single pulses, North American and European soils

**Figure 6:**
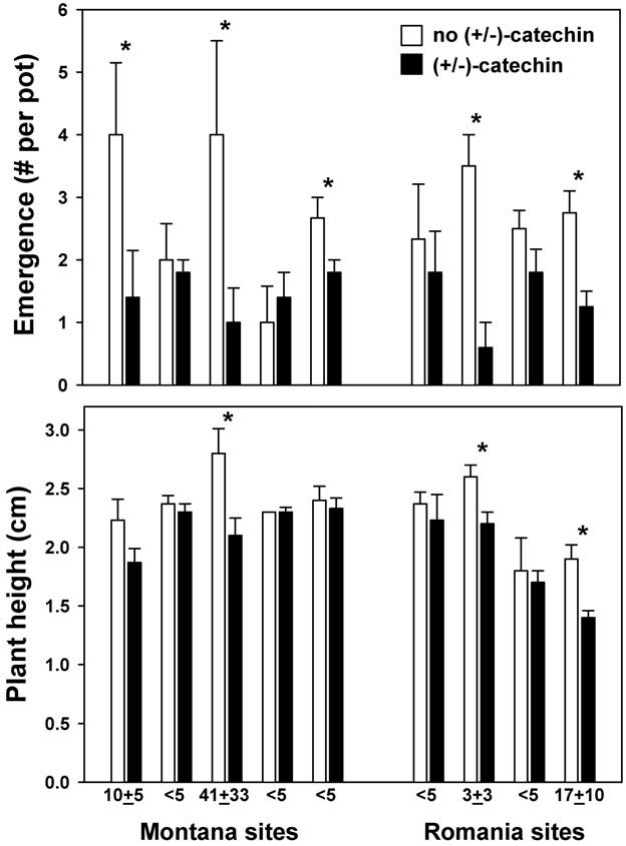
Total emergence and maximum height of *Koeleria macrantha* seedlings grown in soils collected from five sites in Montana and four sites in Romania, and in treatments with and without (±)-catechin. (±)-Catechin had been added to the soil 12 days prior to sowing seeds, and the numbers below the bars show the concentration in the soil two days before adding seeds. Error bars show 1 SE and asterisks denote significant differences between treatments at a particular site as determined by separate t-tests. The analysis from the complete ANOVA model is presented in the results.

**Figure 7:**
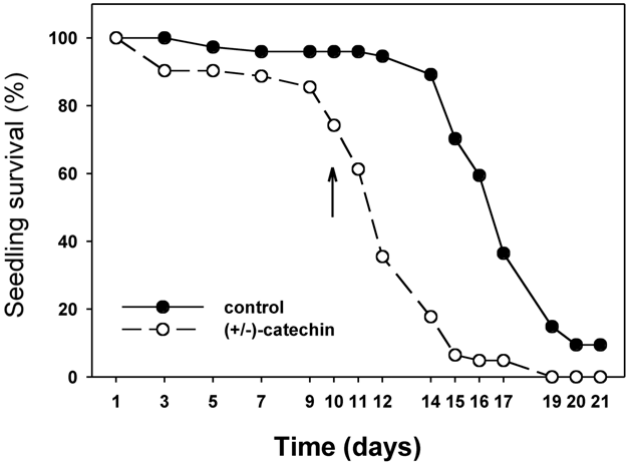
Percent survival of *Koeleria macrantha* seedlings grown in soils collected from five sites in Montana and four sites in Romania, and in treatments with and without (±)-catechin. (±)-Catechin had been added to the soil 12 days prior to sowing seeds, and no (±)-catechin was applied during the time course shown here. Results from all nine sites are combined. The arrow on the graph denotes when watering was stopped, and afterwards seedlings were exposed to increasingly drier soils. Kaplan-Meier survival analysis and Chi-square analyses are presented in the results.

In the fourth experiment with soils from Montana and Romania, when seeds were planted after (±)-catechin concentrations had been allowed to decline for 12 days (see Fig. 3), treatment of the soils with (±)-catechin corresponded with significantly lower seedling emergence, height, and survival (Figs. 6, 7). Planted 12 days after (±)-catechin application, and after soil concentrations had decreased to low or undetectable levels, seedling emergence of *Koeleria* was inhibited significantly in 5 of the 9 soils and seedling height was reduced in 3 of the 9 soils. Using an ANOVA model with untransformed data (the means for each pot), with continent and treatment as fixed variables and site as a random variable, only the effect of (±)-catechin treatment was significant for emergence (F = 64.12; df = 1,54; P = 0.004) and for height (F = 32.55; df = 1,40; P = 0.0011). Furthermore, the survival of *Koeleria* seedlings in the (±)-catechin treatment decreased much faster after being exposed to drought than seedlings in control soils (Fig. 7). Kaplan-Meier Survival Analysis demonstrated significantly lower survival among *Koeleria* seedlings exposed to drought in the (±)-catechin treatment than in the control (Log-rank Chi-square = 7.224; df = 1; P = 0.007; Breslow Chi-square = 3.297; df = 1; P = 0.069; Tarone-Ware Chi-square = 4.703; df = 1; P = 0.030).

#### Single pulse, in situ soils

**Figure 8:**
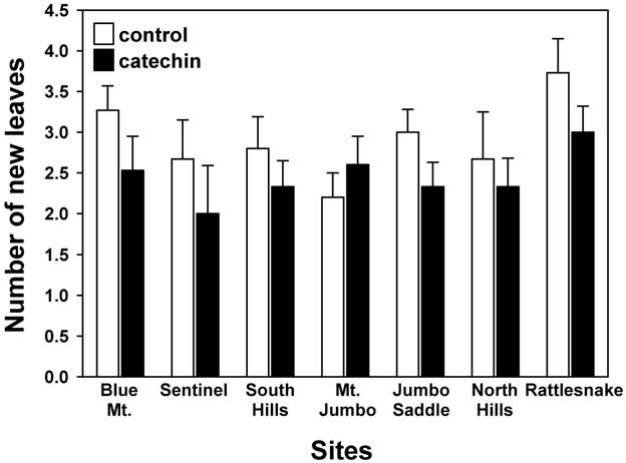
Change in leaf number for *Koeleria macrantha* in the field with and without (±)-catechin injected into rhizosphere soils. Error bars show 1 SE. We tested the effects of (±)-catechin on growth across all sites (n = 15 in each treatment at each site) with a two way ANOVA, with (±)-catechin as a fixed factor and site as a random factor (SPSS 15.0, SPSS, Chicago). ANOVA, F_treatment_ = 8.86; df = 1,196; P = 0.025, F_site_ = 3.09; df = 6,196; P = 0.098).

The mean leaf growth of the control plants in the field experiment was 2.90±0.16 (1 SE) leaves per plant versus 2.45±0.15 for the plants treated with (±)-catechin (Figure 8). A subsample of soils collected immediately after application demonstrated no detectable (±)-catechin. Based on separate t-tests, there was no significant effect of (±)-catechin addition at any single site, but the overall treatment effect was significant (ANOVA, F_treatment_ = 8.86; df = 1,196; P = 0.025, F_site_ = 3.09; df = 6,196; P = 0.098).

## Discussion

Our results demonstrated phytotoxicity for (±)-catechin at low concentrations in Petri dishes and in soils. In some cases we measured toxicity when (±)-catechin concentrations were below the 5 µg g^−1^ detection limit of our protocol. We also measured phytotoxicity in Indian soils amended with organic matter, river sand, and *in situ* field experiments in Montana applying concentrations within the range produced *in vitro* by *C. maculosa* seedlings. Collectively, these results provide a significant step towards understanding the role of catechin in the natural environment and understanding allelopathy in general. A large number of other studies have demonstrated phytotoxicity of (±)-catechin *in vitro* and in sand cultures [16]–[22], [24], [25]–[27], [40], in experiments with field soils [18], [27] and in field applications [6], [23], [27], and our results help to put toxicity into the context of natural soil concentrations (±)-catechin and amounts exuded by seedlings.

We do not know the reason for the disparity between our results for (±)-catechin phytotoxicity (and those cited above that show phytotoxicity) and experiments that have not [25], [28], [29], but there are several possibilities worth considering. First, the rate at which different forms of catechin oxidize could lead to substantial differences if the oxidized forms are not toxic or if other forms are more toxic. Our non-quantified observations suggest that oxidation of (±)-catechin appears to be affected by exposure to light, the presence of seeds in the solution, contaminants in containers, different sources of the water used for the solution, and time. We also found that (±)-catechin was more toxic in some soils than would be estimated from its effects in solutions *in vitro*. This raises the possibility that some chelated forms of (±)-catechin, perhaps with different metals, may be more phytotoxic than the pure form, and we are currently exploring this possibility. Regardless, because (±)-catechin oxidizes and chelates rapidly, applied concentrations are a poor benchmark for phytotoxicity, substantially overestimating the concentration of (±)-catechin required for phytotoxicity *in vitro* and in the field. Furthermore, soil texture modifies allelopathic expression [48] and organic matter may affect the biological effects of compounds by coating metal surfaces and preventing compounds from coming into contact with mineral ions, thus slowing the rate of oxidation [36] or chelation processes. Microbial mineralization or decomposition to non-toxic forms [45], [49] may also affect the dynamics of allelochemicals in soils. For example, *Acinetobacter calcoaceticus* bacteria use catechins as carbon sources [49].

For a number of different soils and experimental conditions we demonstrated phytotoxicity of (±)-catechin at and soil concentrations approaching that detected in the field by Blair *et al.* [28] are far below the pulse reported by Perry *et al.* [30]. Variation in the phytotoxicity of any allelochemical can be affected by differences in extraction methodology, analytical techniques, variation in the rates of chemical degradation, and the vagaries of experimental application procedures [50]. Therefore it is not surprising that substantial variation has been found among experiments. However, it is important to note in this context that we found that known amounts of (±)-catechin experimentally added to soils were dramatically reduced to very low, or even undetectable levels (<5 µg g^−1^) but still produced phytotoxic effects in some soils, including our field tests. It is not clear if “pure” (±)-catechin itself is phytotoxic at such low levels, but *in vitro* experiments suggest this is not the case. We may not have detected all (±)-catechin in our test soils, but our repeated measurements of (±)-catechin dynamics in some soils and comparative extraction protocols demonstrate that we were unlikely to have missed large amounts. On the other hand there might have been phytotoxic effects of chelated forms of catechin or other degradation products as well as (±)-catechin itself. The total phenolic content of soils amended with 0, 340, 680 0r 1020 µg catechin/Indian soil was 3.6±0.6, 16.2±1.62, 23.3±0.8 and 36.1±0.9 µg/g soil, respectively. Research by Furubayashi et al. [25] indicates that the degradation products, tested in one soil type, of the+form of catechin are much less toxic than the pure form *in vitro*, but they used lettuce as a target species, which is unusually resistant to (±)-catechin, relative to many native North American species [22].

Our results also demonstrate the potential for drought to interact with the effects of (±)-catechin (see [51]). Such allelopathy-by-environment interactions are commonly overlooked, but have the potential to cause substantial variation in allelopathic experiments. In summary, our results clearly demonstrate that (±)-catechin has the potential to play an important ecological role in the invasion of *Centaurea maculosa* in North America and support the Novel Weapons Hypothesis.
